# High Sural Nerve Trifurcation: A Rare Anatomical Variation

**DOI:** 10.7759/cureus.30606

**Published:** 2022-10-23

**Authors:** Eng Kee Tan, Azammuddin Alias, Raymond Yeak, Mohd Shahril Jaafar, Nasir M Nizlan

**Affiliations:** 1 Department of Orthopedics and Traumatology, Universiti Putra Malaysia, Serdang, MYS

**Keywords:** trimalleolar ankle fracture, ankle and foot, anatomical variation, trifurcation, sural nerve

## Abstract

The sural nerve is a commonly encountered anatomical structure in foot and ankle surgeries. Knowledge of its location and course is imperative in performing surgeries within its vicinity to avoid neurological deficits. We herein report a rare anatomical variation of the sural nerve where it trifurcates above the level of the lateral malleolus that was discovered in a patient who underwent internal fixation for a trimalleolar ankle fracture with ipsilateral navicular fracture. This study aimed to raise awareness on a unique anatomical variation of the sural nerve in order to reduce the risk of iatrogenic injury.

## Introduction

The sural nerve is a cutaneous sensory nerve originating from the distal third of the gastrocnemius muscles and descends along the posterolateral region of the leg down to the rear aspect of the lateral malleolus, beneath the fibularis tendon sheath. It then bifurcates into the lateral dorsal sensory nerves and the lateral calcaneal branches providing cutaneous innervation of the lateral foot and lower ankle region [1]. Anatomical variations of this nerve and its close proximity to the small saphenous vein, peroneus tendons, and lateral malleolus put it at risk for iatrogenic injuries involving posterolateral surgical approach for ankle reconstructions [2]. Here, we report a case of a patient seen at a tertiary hospital who sustained a trimalleolar right ankle fracture and intraoperatively was found to have a unique sural nerve anatomical variation superior to the lateral malleolus.

## Case presentation

**Figure 1:**
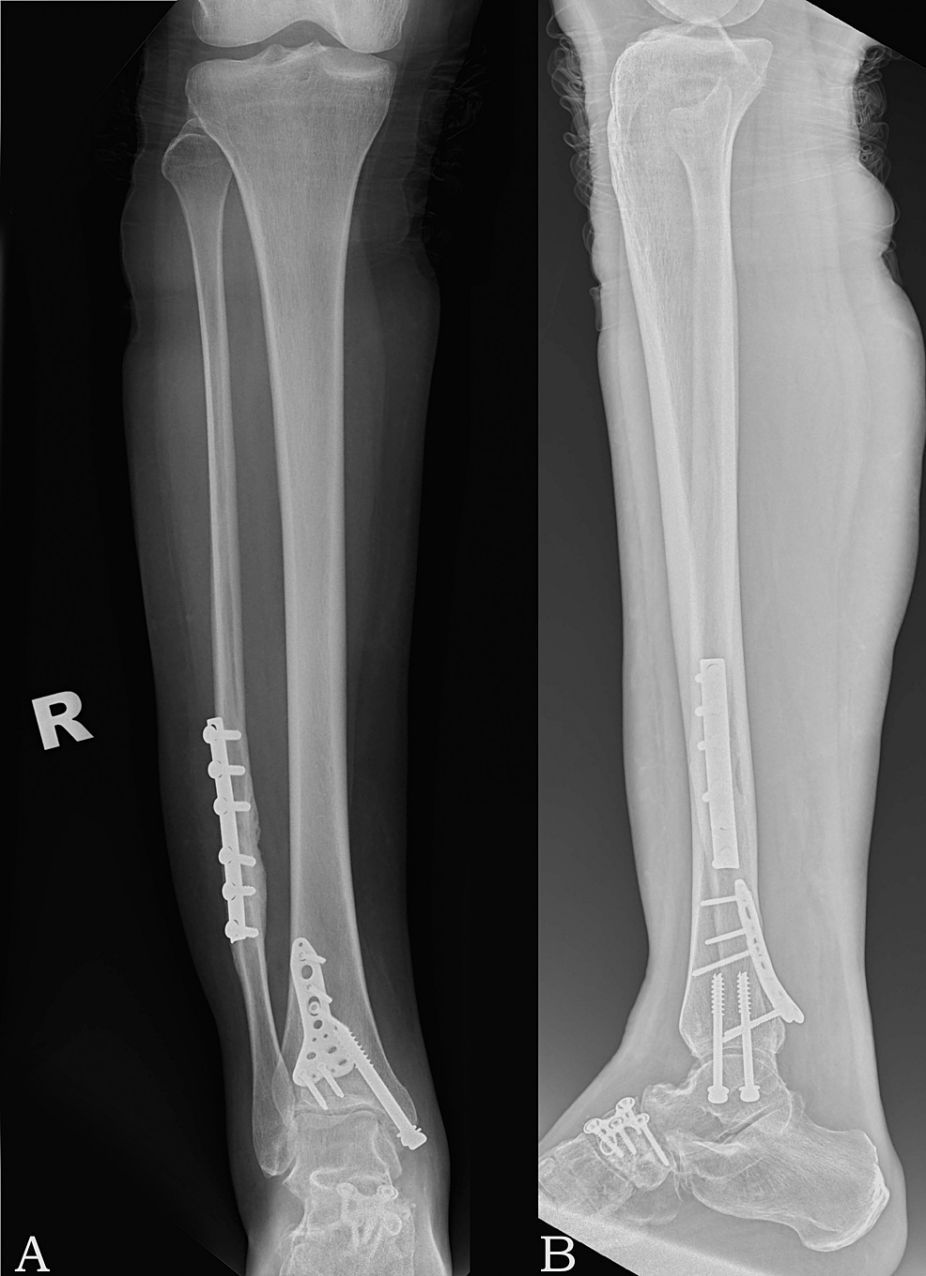
Postoperative tibia and fibula anteroposterior (AP) and lateral radiographs with implants in situ. (A) AP view of the right tibia fibula radiograph and (B) lateral view of the right tibia fibula radiograph.

A 21-year-old Indian female with no known medical illness was admitted for a closed right trimalleolar fracture with ankle subluxation sustained from a motor vehicle accident. Sensation of the right ankle and foot was intact during the presentation. X-rays showed right distal third fibular fracture as part of a trimalleolar fracture with ankle subluxation along with a navicular fracture. Surgery for internal fixation of the fibula and posterior malleolus was performed for her through a posterolateral incision. Locking plates were inserted for the posterior malleolus and navicular fractures while the fibula and medial malleolus were fixed with one-third tubular plate and screw fixation, respectively (Figures 1A, 1B).

**Figure 2:**
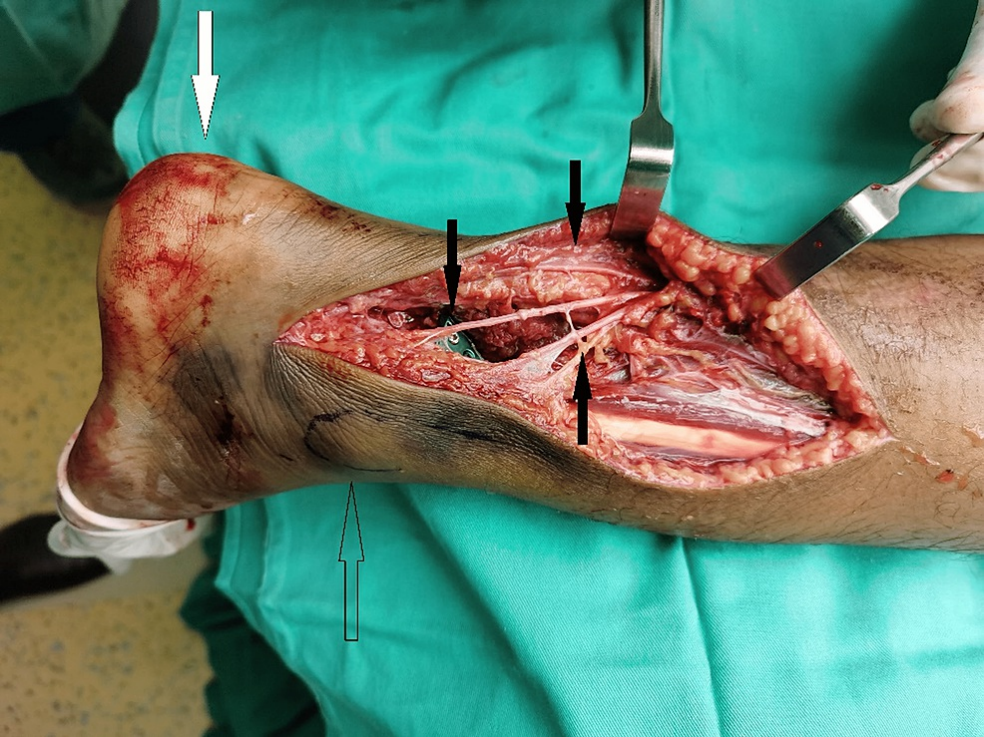
Trifurcation of sural nerve originating superior to lateral malleolus. The image shows trifurcation of sural nerve (black arrows), heel (white arrow), and lateral malleolus (clear arrow).

Intraoperatively, an unusual sural nerve anatomical variation was identified. Sural nerve trifurcation was seen high at the distal third of gastrocnemius muscle with extension of all three branches posterior to the lateral malleolus (Figure 2). Postoperatively, right ankle and foot sensations were intact, and the patient was able to bear weight with no active complaints after one month.

This case highlights the presence of an unusual sural nerve anatomical variation and the potential for iatrogenic injury intraoperatively if the surgeon fails to recognize the diverse anatomical presentations of the sural nerve.

## Discussion

According to orthopedic principles, trimalleolar ankle fractures with intraarticular involvement require internal fixation in order to restore the congruence of the articular surface. The posterolateral fracture fragment and the fibular fracture can be accessed using a single posterolateral approach. For this, the patient is placed in a prone position, and a 10-15 cm incision is made between the Achilles tendon and fibula. During deep dissection in this approach, the surgeon will likely encounter the sural nerve that runs parallel just adjacent to the lateral margin of the Achilles tendon [3].

The sural nerve otherwise known as the short saphenous nerve arises from the tibial nerve and common peroneal nerve, both originating from the sciatic nerve. Common variations of sural nerve consist of merging between the medial sural cutaneous nerve (MSCN) with the peroneal communicating nerve (PCN), continuation of MSCN with non-existent PCN, or the union of MSCN with lateral sural cutaneous nerve (LSCN) [4]. Despite extensive research on the diverse anatomical sources of sural nerve, literature on various tributaries of the sural nerve have been scarce [4]. Well-known branches of the sural nerve include lateral calcaneal, lateral malleolar, and lateral dorsal cutaneous branches. These branches mostly arise from the retromalleolar aspect with the sural nerve itself giving off three branches that extend to the lateral aspect of the heel [5]. Findings from numerous cadaveric dissections have consistently identified tributaries of the sural nerve arising from the posterolateral foot but there are very limited reports describing divisions of the sural nerve superior to lateral malleolus [5].

A trifurcated sural nerve arising proximal to the lateral malleolus is a unique discovery and raises the question of what implications it could have on ankle and foot surgeries involving posterolateral approaches. In this patient, the sural nerve divides into three distinct branches posterior to the distal one-third of gastrocnemius of the right foot. The posterior-most branch descends in close relation to the Achilles tendon as well as the posterior malleolus. Intermediate and lateral branches travel posterior to lateral malleolus and are closely related to peroneus sheath and small saphenous vein. With this distribution and proximity to nearby structures in mind, it is therefore important to be aware of the high risk for iatrogenic injury to the sural nerve during ankle and foot surgeries, especially via posterolateral approach as the nerve branches traverse the incision site as is in this case.

## Conclusions

A comprehensive understanding of the sural nerve anatomy, origin, variations, and tributaries are important for surgical practice. Surgeons should be highly aware of the possible variations in sural nerve formation and branching to avoid iatrogenic injuries intraoperatively. Although the likelihood of encountering multiple tributaries such as in this case is rare, it is crucial to be aware of such possibilities and the need for careful identification of the entirety of sural nerve. It is at significant risk during posterolateral incisions during reconstruction of the ankle, peroneal or Achilles tendon repairs, fracture fixations of the lateral and posterior malleolus. Due to this, we strongly suggest careful isolation and protection of the sural nerve and its tributaries during surgery.
